# Hamstring Graft Diameter and Clinical Outcomes in Anterior Cruciate Ligament Reconstruction: A Retrospective Cohort Study

**DOI:** 10.7759/cureus.103597

**Published:** 2026-02-14

**Authors:** Matteo Denti, Dario Giunchi, Michael-Alexander Malahias

**Affiliations:** 1 Studio Dr. Denti, Clinica Ars Medica, Gravesano, CHE; 2 Department of Orthopaedics and Traumatology, School of Medicine - Frankfurt, Frankfurt, CHE

**Keywords:** anterior cruciate ligament reconstruction, arthroscopy, clinical outcomes, graft diameter, hamstring tendon autograft, knee stability, knee surgery, kt-1000 arthrometer, re-rupture rates, sports traumatology

## Abstract

Purpose: The outcomes of anterior cruciate ligament (ACL) reconstruction can be influenced by multiple factors, including surgical technique, graft type, and fixation methods. While existing literature has extensively examined these variables, less attention has been given to the impact of graft diameter. This study aims to evaluate the effect of hamstring graft diameter on clinical outcomes and recurrence rates following ACL reconstruction, addressing a significant gap in the current research.

Methods: We conducted a retrospective analysis of prospectively collected data from 107 patients who underwent ACL reconstruction with a quadrupled hamstring tendon autograft between 2007 and 2019 at Via Grumo 16, Gravesano, Switzerland. Patients were divided into two groups based on graft diameter: 5-7 mm and ≥8 mm. The primary outcome was knee stability, assessed using the KT-1000 arthrometer at six, 12, and 24 months post-surgery. Re-rupture rates and other clinical outcomes were also evaluated. Statistical analysis was performed using Kaplan-Meier survival curves, Cox regression, and non-parametric tests, with a power analysis conducted to assess the adequacy of the sample size.

Results: No significant differences in knee stability, as measured by KT-1000 scores, were observed between the two groups at any follow-up interval (p > 0.05). Re-rupture rates were also comparable, with no significant association between graft diameter and the risk of re-rupture identified (p > 0.05). The study’s power analysis revealed that our sample size did not reach the 80% threshold, representing a notable limitation.

Conclusion: Our findings suggest that graft diameter may not significantly influence clinical outcomes in ACL reconstruction with hamstring tendons, challenging prior assertions of its critical role. Given the study’s limitations, including its underpowered sample size, further research with larger cohorts is needed to validate these results and guide graft selection strategies.

## Introduction

Anterior cruciate ligament (ACL) reconstruction is one of the most commonly performed orthopedic procedures, yet technical controversies remain, particularly regarding graft selection and its impact on surgical outcomes. ACL injuries represent a substantial epidemiological burden, with hundreds of thousands of cases reported annually worldwide. They affect both elite athletes and the general active population, often resulting in significant functional limitation, time away from sport, and long-term socioeconomic impact. The ideal graft for ACL reconstruction should exhibit biological and mechanical properties that closely replicate those of the native ligament, ensuring optimal stability and long-term function. Current evidence suggests that autografts are generally superior to allografts in young, active patients due to their more favorable healing and biomechanical profiles [1,2]. Among the various autograft options, hamstring tendon grafts are favored for their strength, reduced donor-site morbidity, and overall positive clinical outcomes [3,4].

However, despite these advantages, there remains a higher reported failure rate for hamstring autografts compared to bone-patellar tendon-bone grafts [5]. This has led to a growing interest in understanding the factors that influence graft success, with graft diameter emerging as a key determinant. Specifically, smaller graft diameters have been implicated in increased risk of graft failure, particularly in younger, more active populations [6,7]. The purpose of this study is to investigate the relationship between graft diameter and clinical outcomes, including knee stability and re-rupture rates, after ACL reconstruction using hamstring tendons.

Although larger graft diameters are theoretically associated with improved biomechanical properties, real-world clinical outcomes are not uniformly consistent across published studies. Therefore, the role of graft diameter may be more nuanced than previously assumed, and understanding its true clinical impact requires dedicated investigation.

Our primary aim is to determine whether a larger graft diameter correlates with improved knee stability, as measured by the KT-1000 arthrometer. The secondary aim is to assess the influence of graft size on re-rupture rates over a two-year follow-up period. This study addresses a notable gap in the literature, as previous research has often provided conflicting results or lacked rigorous statistical analyses [8,9]. By exploring this relationship, we seek to contribute to the ongoing discussion and provide evidence-based recommendations for optimizing graft selection.

## Materials and methods

This retrospective study was conducted at Via Grumo 16, Gravesano, Switzerland, and included 107 patients who underwent ACL reconstruction between 2007 and 2019. The study received approval from the IRB by Studio Dr. Denti 007/2023. Exclusion criteria included multiligamentous knee injuries, previous ipsilateral knee surgery, severe lower-limb malalignment requiring correction, incomplete clinical follow-up, or insufficient documentation of graft size or postoperative KT-1000 measurements. Data were collected prospectively and reviewed retrospectively to maintain data integrity. The surgical procedures were performed by a single experienced orthopedic surgeon, while post-operative assessments were conducted by a separate, blinded physician to reduce potential bias.

Patients were categorized into two groups based on graft diameter: Group 1 comprised patients with grafts measuring between 5 and 7 mm, and Group 2 included those with grafts measuring ≥8 mm. The choice of the 7.5 mm cut-off was informed by prior research that identified this threshold as critical for improving clinical outcomes [6,7]. Demographic variables, such as age, BMI, activity level, and concomitant injuries, were recorded to account for potential confounders.

Additional variables known to influence graft success, such as patient age, associated meniscal lesions, and lower-limb alignment, were documented when available, although the study was not powered to analyze each determinant independently.

The surgical protocol involved harvesting and preparing the hamstring tendons through a standard approach. A 3 cm longitudinal skin incision was made at the tibial insertion, followed by meticulous dissection to expose and isolate the gracilis and semitendinosus tendons. The tendons were stripped of muscle fibers, tensioned, and quadrupled to create the final graft. Graft diameter was measured using progressive sizers with 0.5 mm accuracy [8].

Post-operatively, patients followed a structured rehabilitation program that allowed for immediate weight-bearing with quadriceps activation. Return to sport was generally advised at nine months, contingent on clinical evaluation, isokinetic testing, and MRI confirmation of graft ligamentization. Clinical assessments were performed at six, 12, and 24 months post-surgery. The KT-1000 arthrometer was used to measure knee stability, with a side-to-side difference of >3 mm defined as a failure [5]. Re-rupture rates and knee function outcomes were also evaluated.

Statistical analysis

Statistical analyses were conducted using R software (version 4.2.1, R Foundation for Statistical Computing, Vienna, Austria, https://www.R-project.org/). Survival curves were generated using the Kaplan-Meier method, and Cox regression models were applied to assess the influence of variables such as graft size, age, and BMI on the hazard of re-rupture. Fisher’s exact test and chi-square tests for trend were used for categorical data, while the Mann-Whitney U test and Spearman’s correlation were employed for non-parametric data. A power analysis was conducted to ensure the sample size was adequate, with a threshold of 80% considered statistically robust.

## Results

Last KT-1000

No differences were observed in terms of the last KT-1000 score (at least 12 months postoperatively) between the two groups. Indeed, trends between proportions of growing KT-1000 score were similar between groups (p = 0.257), as well as the fractions of subjects with KT-1000 ≥3 mm at the last follow-up (1/38 in >7.5, 2/69 in <7.5; p = 0.999). The distribution of KT-1000 values at the final follow-up is shown in Table 1.

**Table 1:** Last KT-1000 values

Last KT-1000	>7.5	<7.5
<0 mm	5	8
0-1 mm	25	37
1-2.5 mm	7	22
>2.5 mm	1	2

Re-rupture rates

**Figure 1:**
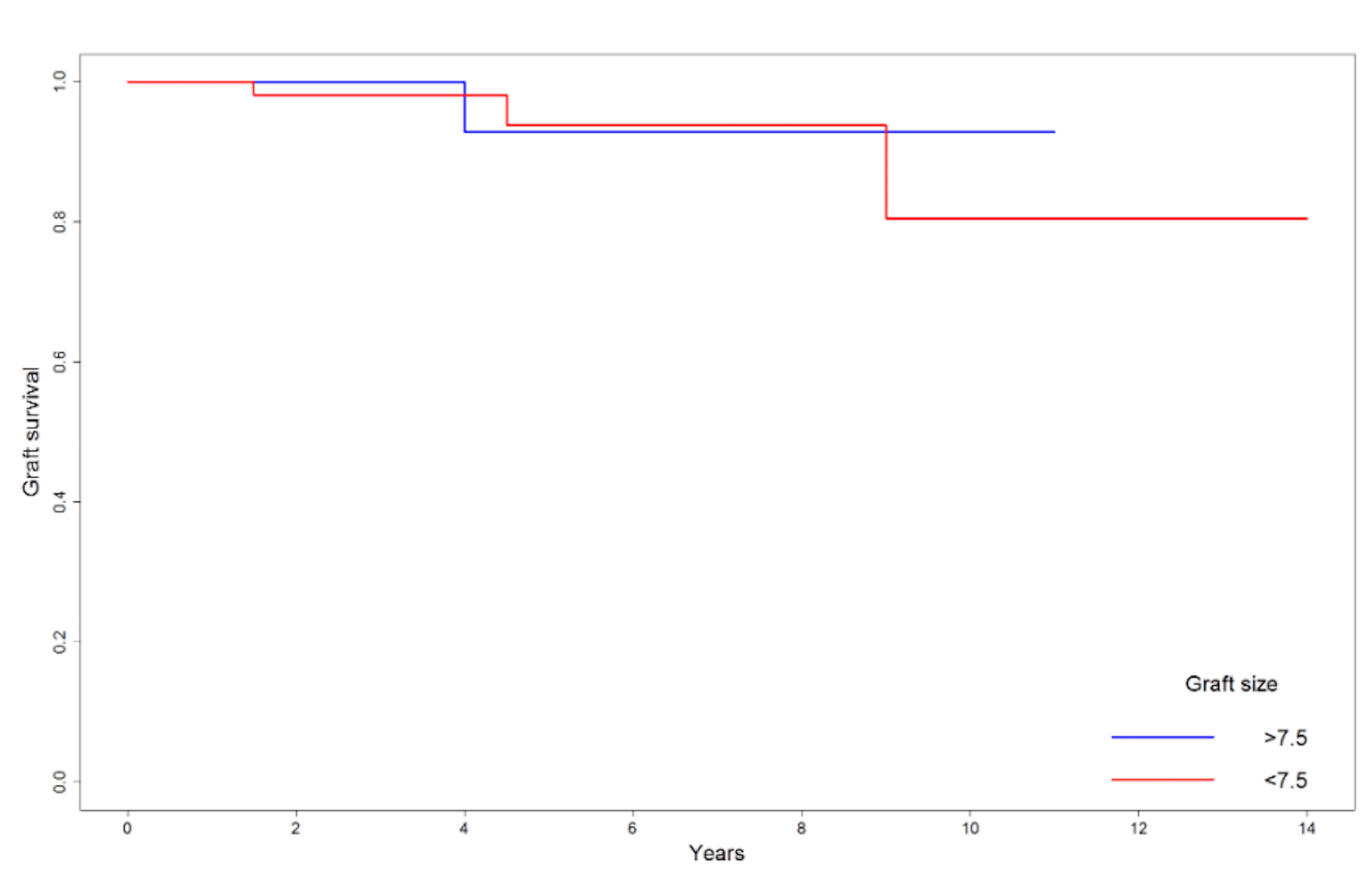
Survival curve of graft in the two study groups (graft size >7.5 and <7.5). Image created by the authors with R (R Foundation for Statistical Computing, Vienna, Austria)

The re-rupture rates were comparable between groups, with two out of 69 cases in the smaller graft group and one out of 38 cases in the larger graft group (p = 0.999). Cox regression analysis, performed with graft size treated as both a continuous and categorical variable, revealed no significant associations with the hazard of re-rupture (continuous: p = 0.925; dichotomous: p = 0.665) [8]. The comparison of graft survival between the two groups is illustrated in Figure 1.

KT-1000 at six months

No differences were observed at six months in KT-1000 comparing subjects with graft size < and >7.5. One patient in <7.5 and 3 in >7.5 showed high KT-1000, but the proportions were not significantly different (p = 0.127). Chi-square for trends showed similar proportions between the two groups in growing categories of KT-1000 (p = 0.919). Moreover, considering KT-1000 as numerical values, no differences emerged between the two groups (p = 0.731), and no correlation between KT-1000 values and numeric graft size emerged (p = 0.726). The detailed distribution of KT-1000 values at six months is presented in Table 2.

**Table 2:** KT-1000 at six months

KT-1000 six months	>7.5	<7.5
<0 mm	4	4
0-1 mm	25	48
1-2.5 mm	4	12
>2.5 mm	3	1

KT-1000 at 12 months

One patient in <7.5 and 3 in >7.5 showed high KT-1000 at 12 months, but the proportions were not significantly different (p = 0.662). Chi-square for trends showed similar proportions between the two groups (p = 0.995). Moreover, considering KT-1000 as numerical values, no differences emerged between the two groups (p = 0.775), and no correlation between KT-1000 values and numeric graft size emerged (p = 0.627). The full breakdown of KT-1000 values at 12 months is reported in Table 3.

**Table 3:** KT-1000 at 12 months

12 months	<7.5	>7.5
<0 mm	3	7
0-1 mm	25	40
1-2.5 mm	3	12
>2.5 mm	3	3

KT-1000 at 24 months

At two years, even if fewer subjects were present (n = 51), the graft size appeared to be associated with KT-1000.

Indeed, even if no difference was observed in terms of fraction of patients with KT-1000 >2.5 mm (p = 0.523) between <7.5 (2/30) and >7.5 (0/19), chi-square for trends showed nearly significant differences between the two groups (p = 0.086, see table). In addition, considering KT-1000 as numerical values, differences emerged between the two groups (>7.5: median 0, interquartile range 0-0; <7.5: median 0, interquartile range 0-1; p = 0.028) and a weak negative correlation was observed between KT-1000 values and numeric graft size, close to significance (r = -0.260, p = 0.065). The distribution of KT-1000 values at 24 months is summarized in Table 4.

**Table 4:** KT-1000 at 24 months

24 months	>7.5	<7.5
<0 mm	2	3
0-1 mm	17	22
1-2.5 mm	0	5
>2.5 mm	0	2

Power analysis

Analyses were performed using R software (v4.2.1, R Core Team, Vienna, Austria). Survival was assessed by Kaplan-Meier curve with log-rank test, and Cox regression models were used to test the influence of different variables (graft size, age, BMI, gender, and presence of other lesions) on the hazard of re-rapture. Fisher’s exact test was used to assess differences between proportions of dichotomous variables in the two study groups, while chi-square tests for trend were used to assess different proportions of ordered categorical variables between the groups. When KT-1000 was used as numeric values, a non-Gaussian distribution was assumed, and a non-parametric hypothesis test was applied (Mann-Whitney U test). For the same reason, correlations were assessed using Spearman’s method. P values <0.05 were considered statistically significant

A post-hoc power analysis was conducted to compare the rate of re-reputures between groups. Assuming the observed rates in the study cohorts (2.9% and 2.6%) are accurate, a sample size of 59,182 patients per group would be required to achieve a significance level of α = 0.05 and a statistical power of 80%. For achieving 80% power with effect sizes categorized as small (0.3), medium (0.5), and large (0.8), and given 69 subjects in the small graft group, the large graft group would need 175, 63, or 15 patients, respectively.

## Discussion

This study explores the influence of graft diameter on clinical outcomes in ACL reconstruction using hamstring autografts. Contrary to some well-established notions in the literature, our results demonstrate no significant differences in knee stability or re-rupture rates between smaller (<7.5 mm) and larger (≥8 mm) grafts. These findings invite a reassessment of the emphasis placed on graft size in clinical decision-making, which has long been considered a crucial determinant of surgical success [6,7,8,9].

The role of graft diameter in ACL reconstruction has been debated extensively. Magnussen et al. found that smaller grafts (<8 mm) were associated with a significantly higher failure rate, especially in younger patients, driving the consensus that larger grafts offer greater protection against re-injury [6]. Snaebjörnsson et al. similarly reported a reduced risk of revision with every 0.5 mm increase in graft size, emphasizing diameter as a critical factor [7,8]. Tang et al. confirmed this trend in the Chinese population [9]. Jeffers et al. also reported high return-to-play and low reinjury rates using quadrupled hamstring autografts, supporting graft adequacy beyond diameter alone [10,11]. Ramkumar et al. highlighted anthropometric predictors of hamstring graft size, reinforcing the multifactorial determinants of graft adequacy [12].

One reason for our differing results may lie in the complexity of ACL biomechanics. While graft size undeniably contributes to structural integrity, factors such as precise tunnel placement, graft tensioning, and the biomechanical properties of the knee joint may have an equally or more significant influence on outcomes [5,8,12]. While graft diameter may contribute to biomechanical integrity, it is well established that accurate anatomic tunnel placement remains the primary determinant of ACL reconstruction success. Our findings should therefore be interpreted within this broader hierarchy of surgical factors. Furthermore, patient-related characteristics, including activity level, age, and body mass index (BMI), likely interplay with graft performance, complicating the simplistic view that “bigger is always better.”

Our study stands out for its methodological rigor. All surgical procedures were performed by a single, highly experienced orthopedic surgeon, minimizing variability in technique. Additionally, outcomes were evaluated by an independent, blinded assessor, enhancing the reliability of our data. By challenging the entrenched belief that graft diameter is a paramount factor, our work invites further exploration into what truly optimizes ACL reconstruction [5,8,12].

Importantly, our results should not be interpreted as encouragement to deliberately select smaller grafts. Clinical decision-making must continue to consider patient anatomy, activity demands, and optimal surgical technique, including precise tunnel placement.

Despite its strengths, our study is not without limitations. The sample size, although adequate for the primary comparison, did not allow for sufficiently powered subgroup analyses (e.g., by age, activity level, or meniscal status). Future multicenter studies with larger cohorts are required to explore these variables in greater depth. Our retrospective design, while using prospectively collected data, carries inherent biases, and our findings stem from a single-institution study, potentially impacting generalizability. Moreover, our analysis did not account for every conceivable confounding factor, such as specific rehabilitation protocols that might influence outcomes. To build on our findings, future studies should employ larger, multi-center cohorts and consider additional variables such as pre-operative imaging predictors [13,14], intraoperative technical adjustments [15], and inter-observer reliability in graft sizing [16].

Additional subgroup analyses were considered; however, given the sample size of 107 patients, further stratification would have resulted in underpowered comparisons. For this reason, we limited the analysis to the primary research question.

These results encourage clinicians to adopt a more holistic view when planning ACL reconstruction. Rather than prioritizing graft size alone, a tailored approach that incorporates patient anatomy, activity level, and surgical precision may yield better long-term outcomes.

## Conclusions

Our findings challenge the prevailing emphasis on graft diameter as the primary determinant of ACL reconstruction success. While our data suggest that size may not be as crucial as once thought, they underscore the multifaceted nature of successful ACL surgery. These findings contribute to a more nuanced understanding of graft diameter within the broader context of ACL reconstruction, while reaffirming the importance of individualized graft selection and meticulous anatomic technique.

## References

[1] Lin KM, Boyle C, Marom N, Marx RG (2020). Graft selection in anterior cruciate ligament reconstruction. Sports Med Arthrosc Rev.

[2] Duchman KR, Lynch TS, Spindler KP (2017). Graft selection in anterior cruciate ligament surgery: who gets what and why?. Clin Sports Med.

[3] Chen L, Cooley V, Rosenberg T (2003). ACL reconstruction with hamstring tendon. Orthop Clin North Am.

[4] Ibrahim SA, Al-Kussary IM, Al-Misfer AR, Al-Mutairi HQ, Ghafar SA, El Noor TA (2005). Clinical evaluation of arthroscopically assisted anterior cruciate ligament reconstruction: patellar tendon versus gracilis and semitendinosus autograft. Arthroscopy.

[5] Persson A, Fjeldsgaard K, Gjertsen JE, Kjellsen AB, Engebretsen L, Hole RM, Fevang JM (2014). Increased risk of revision with hamstring tendon grafts compared with patellar tendon grafts after anterior cruciate ligament reconstruction: a study of 12,643 patients from the Norwegian Cruciate Ligament Registry, 2004-2012. Am J Sports Med.

[6] Magnussen RA, Lawrence JT, West RL, Toth AP, Taylor DC, Garrett WE (2012). Graft size and patient age are predictors of early revision after anterior cruciate ligament reconstruction with hamstring autograft. Arthroscopy.

[7] Snaebjörnsson T, Hamrin Senorski E, Ayeni OR (2017). Graft diameter as a predictor for revision anterior cruciate ligament reconstruction and KOOS and EQ-5D values: a cohort study from the swedish national knee ligament register based on 2240 patients. Am J Sports Med.

[8] Snaebjörnsson T, Hamrin-Senorski E, Svantesson E, Karlsson L, Engebretsen L, Karlsson J, Samuelsson K (2019). Graft diameter and graft type as predictors of anterior cruciate ligament revision: a cohort study including 18,425 patients from the Swedish and Norwegian national knee ligament registries. J Bone Joint Surg Am.

[9] Tang SP, Wan KH, Lee RH, Wong KK, Wong KK (2020). Influence of hamstring autograft diameter on graft failure rate in Chinese population after anterior cruciate ligament reconstruction. Asia Pac J Sports Med Arthrosc Rehabil Technol.

[10] Jeffers KW, Shah SA, Calvert DD (2022). High return to play and low reinjury rates in National Collegiate Athletic Association Division I football players following anterior cruciate ligament reconstruction using quadrupled hamstring autograft. Arthroscopy.

[11] Matzkin E (2022). Editorial commentary: large-diameter quadrupled hamstring autografts are an acceptable option for National Collegiate Athletic Association Division I college football players: we must challenge our comfort zone to be successful in the end zone. Arthroscopy.

[12] Ramkumar PN, Hadley MD, Jones MH, Farrow LD (2018). Hamstring autograft in ACL reconstruction: a 13-year predictive analysis of anthropometric factors and surgeon trends relating to graft size. Orthop J Sports Med.

[13] Leiter J, Elkurbo M, McRae S, Chiu J, Froese W, MacDonald P (2017). Using pre-operative MRI to predict intraoperative hamstring graft size for anterior cruciate ligament reconstruction. Knee Surg Sports Traumatol Arthrosc.

[14] Conte EJ, Hyatt AE, Gatt CJ Jr, Dhawan A (2014). Hamstring autograft size can be predicted and is a potential risk factor for anterior cruciate ligament reconstruction failure. Arthroscopy.

[15] Cruz AI Jr, Fabricant PD, Seeley MA, Ganley TJ, Lawrence JT (2016). Change in size of hamstring grafts during preparation for ACL reconstruction: effect of tension and circumferential compression on graft diameter. J Bone Joint Surg Am.

[16] Dwyer T, Whelan DB, Khoshbin A (2015). The sizing of hamstring grafts for anterior cruciate reconstruction: intra- and inter-observer reliability. Knee Surg Sports Traumatol Arthrosc.

